# Characteristics of type 1 diabetes mellitus in children and adolescents with Down's syndrome in an admixed population

**DOI:** 10.20945/2359-3997000000365

**Published:** 2021-04-29

**Authors:** Débora Martins Ferreira Pessoa, Nara Lívia Rezende Soares da Paz Oliveira, Giuliane de Santana Dantas, Vania de Fátima Tonetto Fernandes, Renata Maria de Noronha, Luis Eduardo Calliari

**Affiliations:** 1 Irmandade da Santa Casa de Misericórdia de São Paulo Departamento de Pediatria São Paulo SP Brasil Serviço de Endocrinologia Pediátrica, Departamento de Pediatria, Irmandade da Santa Casa de Misericórdia de São Paulo, São Paulo, SP, Brasil; 2 Irmandade da Santa Casa de Misericórdia de São Paulo Departamento de Pediatria São Paulo SP Brasil Departamento de Pediatria, Irmandade da Santa Casa de Misericórdia de São Paulo, São Paulo, SP, Brasil; 3 Hospital Infantil Darcy Vargas Departamento de Endocrinologia Pediátrica São Paulo SP Brasil Departamento de Endocrinologia Pediátrica, Hospital Infantil Darcy Vargas, São Paulo, SP, Brasil

**Keywords:** Down syndrome, diabetes mellitus type 1, autoimmunity, child, adolescent

## Abstract

**Objective:**

People with Down's syndrome (DS) have a higher risk of developing type 1 diabetes mellitus (T1D) and may have specific clinical features compared to T1D patients without DS. This study evaluated the clinical and laboratory aspects of T1D in children and adolescents with DS in an admixed population.

**Subjects and methods:**

A case-control study comparing patients with T1D and DS (T1D+DS) to patients with T1D without DS (T1D controls) from two tertiary academic Hospitals in São Paulo, Brazil.

**Results:**

The sample consisted of 9 patients with T1D+DS and 18 T1D age and sex-matched controls. Anti-glutamic acid decarboxylase 65 antibodies were positive in 7/7 of the 9 T1D+DS patients, confirming the presence of diabetes autoimmunity in this group. Mean age at diagnosis of T1D was 4.9 ± 3.9 years in the T1D+DS group and 6.4 years ± 3 in the T1D control group; early diagnosis (<2 years old) occurred in three T1D+DS patients but only in one T1D control patients, both suggesting lower age of diagnosis in T1D+DS group, although without statistical significance (p = 0.282 and p = 0.093, respectively). The T1D+DS group presented lower total insulin dose (0.7 IU/kg/day ± 0.2) and HbA1c (7.2% ± 0.6) than the control group (1.0 IU/kg/day ± 0.3 and 9.1% ± 0.7, respectively) (p = 0.022 and p = 0.047, respectively).

**Conclusion:**

We confirmed the autoimmune etiology of diabetes in people with DS in this admixed population. T1D+DS patients developed diabetes earlier and achieved better metabolic control with a lower insulin dose than T1D controls. These findings are in agreement with previous studies in Caucasian populations.

## INTRODUCTION

Down's syndrome (DS), first described in 1886, is a genetic disorder caused by the presence of a third copy of chromosome 21 (1). It is the most common chromosomal abnormality (2), with an incidence 1:700 to 1:1,100 live births (3,4), and presents with classical facial characteristics, multiple malformations, intellectual disability, and immune and endocrine dysfunction (2,3). Hashimoto's thyroiditis, Graves' disease, and type 1 diabetes mellitus (T1D) are the most common DS-associated endocrine autoimmune diseases (4).

T1D is one of the most frequent chronic diseases in childhood (5). Its incidence has increased, especially in the population under five years old (6,7). In Brazil, the incidence of T1D is 8 per 100,000 children under 15 years of age per year (8). Although the prevalence of both T1D in people with DS and DS in T1D patients is expected to increase, the published absolute numbers and cohorts are relatively small (3,9). Moreover, it has been suggested in the literature that T1D may have different clinical features in people with DS compared to patients without this syndrome (5,9). However, all the studies so far have been in Caucasian populations, and we found no studies coming from admixed populations. We, therefore, performed this study in an admixed sample to evaluate the clinical and epidemiological aspects of T1D in children and adolescents with DS in this population.

## SUBJECTS AND METHODS

A case-control study comparing patients with T1D and DS (T1D+DS group) to patients with T1D without DS (T1D controls) from two tertiary academic Hospitals in Sao Paulo, Brazil – *Santa Casa de Misericórdia de São Paulo* and *Hospital Infantil Darcy Vargas*. The study recruited patients from the Pediatric Diabetes Outpatient Clinic of both centers and included patients diagnosed with DS and T1D aged 18 years old or under. All 9 patients with T1D+DS followed in both clinics were eligible for the study, all families accepted to participate and were included in the study. A control group included patients with T1D but without DS, paired by sex and age, in a proportion of 2:1 (two T1D controls to one T1D+DS patient). These control patients were chosen at random from patients coming for regular consultations in the outpatient clinic, when they could be matched by sex and age with the study group. The diagnosis of T1D in the control group was based on clinical criteria compatible with the International Society for Pediatric & Adolescent Diabetes (ISPAD) definition – chronological age at diagnosis, typical symptoms, presence or absence of diabetic ketoacidosis (DKA), insulin therapy from diagnosis, and, in some cases, beta-cell autoantibodies (10).

The analyzed data were age at diabetes diagnosis, current age, gender, puberty stage, stature, body mass index (BMI), insulin dose, glycated hemoglobin (HbA1c) levels in the preceding year, measured by Turbidimetric Immunoassay (reference range 4-6%), DKA at diagnosis, presence of beta-cell autoantibodies (anti-glutamic acid decarboxylase 65 [anti-GAD] and *islet* cell antibodies [ICA]), and thyroid autoantibodies. Data were obtained from existing medical records and recorded using a specific protocol (Appendix 1) after an Informed Consent (Appendix 2) and an Assent Form (Appendix 3) had been signed by the patients and/or the patient's parents or legal representative.

Data were recorded on a computer database and analyzed by SigmaStat version 3.5 (Systat Software Inc. Chicago, IL). All collected data were compared between T1D and T1D+DS groups. Descriptive results were presented as the mean ± SD and median for numerical variables. Categorical variables are shown as absolute numbers (percentage). Comparisons were made by unpaired t-test or ANOVA followed by Tukey test for numerical variables, as appropriate. Numerical variables without parametric distribution were analyzed by Kruskal-Wallis, one-way ANOVA on Ranks, or Mann-Whitney Rank Sum Test. Nominal variables were compared by Chi-square or Fisher exact test, as appropriate. A p-value < .05 was considered statistically significant. The study was approved by the Ethical Committees (87542618.2.00005479) of the *Irmandade da Santa Casa de Misericórdia de São Paulo* and *Hospital Infantil Darcy Vargas*.

## RESULTS

The sample consisted of 27 patients, 9 (33.3%) in the T1D+DS group, and 18 (66.7%) in the T1D Control group. The pairing of the groups was satisfactory, as shown in Table 1. The age at T1D diagnosis was numerically lower in the T1D+DS group 4.9 ± 3.9 years than in the controls, 6.4 ± 3 years, although with no statistical significance; p = 0.282. There were three patients (33%) in T1D+DS group with T1D diagnosed before the age of 2 years, and only one (5.5%) in the control group; p = 0.093. Ketoacidosis was present at diagnosis of T1D in five (55.5%) patients in the study group and ten (55.5%) in the control group; p = 0.861.

**Table 1:** Comparison of the clinical and laboratory profile of the groups

	T1D+DS	T1D Controls	P
N = 27 patients	9	18	
Age – years (SD)	9.7 (3.1)	9.6 (3.0)	0.991
Gender (M/F)	4/5	8/10	1.000
Puberty – n (%)	4 (44.4%)	10 (55.5%)	0.695
Age at diagnosis – years (SD)	4.9 (3.9)	6.4 (3.0)	0.282
Age at diagnosis < 2 years old - n (%)	3 (33%)	1 (5.5%)	0.093
Height Z – SDS	-2.71 (1.1)	-0.22 (0.9)	<0.001
BMI Z – SDS	0.4 (–0.05 to 0.9)	0,3 (–0.02 to 0.9)	0.979
DKA at diagnosis – n (%)	5 (55.5%)	10 (55.5%)	0.861
HbA1c – % (SD)	7.2 (0.6)	9.1 (2.0)	0.047
Total insulin – IU/kg/day (SD)	0.7 (0.2)	1.0 (0.3)	0.022

Data are shown as mean ± SD and median (min and max range) for numerical variables. Categorical variables are shown as absolute numbers (percentage). Abbreviations: BMI, body mass index; SDS, standard deviation score; DKA, diabetic ketoacidosis; HbA1c, glycated hemoglobin.

As expected, the Z score for height was lower in the T1D+DS group (-2.7 ± 1.1) compared to the T1D control group (-0.2 ± 0.9); p < 0.001. The body mass index (BMI) standard deviation score (zBMI) was similar in both groups, 0.4 (-0.05 to 0.9) in T1D+DS and 0.3 (-0.02 to 0.9) in the T1D controls; p = 0.979.

Anti-glutamic acid decarboxylase (anti-GAD) antibodies had been measured in seven of nine patients in the T1D+DS group and were positive in all seven. The tests were not performed in the other two patients. Islet cell autoantibodies (ICA) had been measured in six patients and were negative in all of them (two patients were lost in follow-up, and one had not been tested for ICA).

Regarding the evaluation of glycated hemoglobin (HbA1c) during the preceding year, the study group presented lower mean values (7.2% ± 0.6) compared with the control group (9.1% ± 0.7); p = 0.047. Total insulin dose was lower in the T1D+DS group (0.7 IU/kg/day ± 0.2) than in the T1D control group (1.0 IU/kg/day ± 0.3); p = 0.022. Thyroid antibodies had also been measured in all patients in both groups and were positive in six (66%) patients in the T1D+DS group and three (16%) patients in the control group; p = 0.026.

## DISCUSSION

According to the World Health Organization (WHO) current classification of diabetes mellitus, DS is placed under the topic “Other genetic syndromes sometimes associated with diabetes” (11) and is considered the genetic syndrome most commonly associated with T1D (4,12,13). A population study carried out in Denmark showed that among children and adolescents with DS, the prevalence of T1D is four times higher than in the general population (0.7% vs. 0.17%) (9). Interestingly, although this association would be expected to be relatively frequent given these figures, the number of patients evaluated in the literature is very low (9,14). To increase our sample size before starting this study, we contacted other reference centers for T1D, looking for patients with this association, but they had no patients with this association and we succeeded in including only one more center, which helped us to reach a total of nine patients. This number is still not high, but except in one study using a longitudinal follow-up database, the *Diabetes-Patienten-Verlausfsdaten* (DPV) (3), which had a sample of 159 individuals up to the age of 20 with DS and T1D, all the other published cohorts have limited numbers. As an example, a prevalence study from Denmark included all individuals with DS born between 1981 and 2000 in the country (1,151) and all T1D patients (2,094) born during the same period and could find only eight patients with T1D+DS (9). Due to the lack of an extensive database in Brazil, it is not easy to include more centers in such a study.

In our study, the mean age at diagnosis of T1D was lower in patients with T1D+DS compared to the controls without DS, although it did not reach statistical significance. This was very similar to the Danish study, where T1D+DS patients had a mean age of diabetes diagnosis of six years old versus eight in the general T1D patients, which also did not reach significance (9). The German/Austrian study using the DPV group did not find this difference (T1D+DS 8.21 years vs. 8.42 T1D controls) but found that 18.3% of the T1D+DS patients had been diagnosed before three years of age, against 6.4% in the T1D control group (3). Data from studies in the United Kingdom and Austria/Germany showed a greater prevalence of T1D diagnosis in people with DS below the age of two (respectively 18.9 and 22%) than in the general population (respectively 6.4 and 7%) (3,9,15). These results coming predominantly from Caucasian population data are similar to ours, since we found a prevalence of 33.3% of patients with diagnosis of T1D at the age of two years old or under in the T1D+DS group, against 5.5% in the T1D control group.

Patients with T1D+DS tend to be treated with low insulin doses and with simpler regimens, requiring fewer applications a day (3,13). The administration of fewer injections provides a convenient way to deal with patients with different levels of disability and who depend on others for the medication (13). Although this regimen is often associated with sub-ideal glycemic control, in diabetic patients with DS, the level of HbA1c is comparable, or even lower, compared to patients without the syndrome (3,13). In this study, patients with T1D+DS used a lower dose of insulin and achieved better glycemic control than the T1D controls (0.7 vs. 1.0 IU/kg/day; p = 0.022; HbA1c 7.2% vs. 9.1%; p = 0.047, respectively). These findings are consistent with data from the largest study on T1D and DS, the DPV study (3), and other studies (13,16). The need for a lower dose of insulin, associated with better glycemic control, suggests that diabetes in people with DS may be associated with less beta-cell function loss. The simpler lifestyle and the acceptance of the routine could also explain the better metabolic control in these patients (3).

In our patients, two types of beta-cell autoantibodies had been tested, anti-GAD and ICA, the first one was positive in all patients, and the second was negative in all patients tested. These results confirmed the autoimmune etiology of diabetes in DS. As observed in our study, previous studies evaluated the presence of autoantibodies related to diabetes in people with DS and found a high anti-GAD frequency positivity (66% and 69%) (3,17). In another study, carried out in the United Kingdom, two or more markers of autoimmunity against beta cells were present in 6 out of 106 children with DS, showing statistical significance when compared with patients without the syndrome (5.7% vs. 0.45%; p < 0.01) (14). Also, in the same study, anti-GAD levels in people with DS were particularly high in all the samples, with values above the 99th percentile (14). These findings could be interpreted as an indication that anti-GAD is the most persistent antibody (18), or they could open up the possibility of a specific kind of autoimmunity in people with DS. A large multicenter study investigated the human leukocyte antigen (HLA) class II genotype in four groups – healthy control subjects, DS, T1D+DS, and T1D patients. The authors found diabetes-associated HLA class II haplotypes in children with T1D+DS, showing that, in this group of children, HLA is the same as in T1D in the general population (14). The same group, with a larger cohort of patients, did not find the same results years later but confirmed that autoimmunity was still present in people with DS and diabetes (17). These observations taken together suggest that chromosome 21 may also play a role in the polygenic penetrance of T1D in DS, which would not be surprising given the finding of a susceptibility gene, 21q21.11-q22.3, in Danish families (19).

The similarity of the results regarding diabetes autoimmunity among such different genetic backgrounds as European Caucasians and a Brazilian heterogeneous population could be at least partially explained by the findings that the Brazilian population, although highly mixed, is still subject to a major contribution from European ancestry. Previous studies have suggested that the disease risk alleles for T1D come mostly from Europe, and a previous analysis of a Brazilian pediatric population suggested that there was no association between glycemic control and genomic ancestry or self-reported color race (20,21).

All our patients, in both groups, had their thyroid antibodies also tested, considering that thyroiditis is one of the most common autoimmune disorders in DS, with a risk approximately 9.4 higher in people with the syndrome (22), and it is the comorbidity most frequently associated with T1D (4). We found these antibodies positive in 66% of the patients in the study group and 16% in the control group (p < 0.05), confirming the high frequency of the association between T1D and thyroiditis in DS. Other studies that also analyzed this association found similar results, the highest being 74% of thyroid disease in people with DS (3,16,17).

Limitations of the study are mainly related to the sample size, which was restricted due to the small number of patients with this association (T1D+DS) and to the lack of an extensive database in Brazil to collect this data. Fortunately, we managed to gather two large university hospitals in Sao Paulo to include patients. We observe that this difficulty in recruiting patients is also found by other researchers.

In conclusion, to the best of our knowledge, this is the first study of the association between T1D and DS in an admixed population. Diabetes autoimmunity was confirmed in patients with this association and, in comparison with T1D controls, patients with T1D+DS developed diabetes earlier, used a lower insulin dose and achieved better metabolic control. The results were similar to those described in Caucasian populations.

## APPENDIX 1 Questionnaire

### GENERAL INFORMATION

[Table t2]

**Table t2:**

Name:
Date of birth:
Age:
Sex:
Telephone number:
Address:

### DIABETES HISTORY

1. Age at diagnosis of type 1 diabetes mellitus (T1D):
2. Was ketoacidosis present at diagnosis of T1D? ( ) yes ( ) no

### CURRENT DATA

1. Weight:
2. Height:
3. Tanner stage:
4. Which insulin are you currently using?
  ( ) NPH
  ( ) Glargine
  ( ) Degludec
  ( ) Regular
  ( ) Lispro
  ( ) Glulisine
  ( ) Aspart
5. What is the total daily dose?

### LABORATORY TESTS

1. Glycated hemoglobin (2018):
2. Beta cell autoantibodies:
  - Anti-GAD:
  - ICA:
3. Thyroid antibodies:

## APPENDIX 2 INFORMED CONSENT FORM

### Characteristics of type 1 diabetes mellitus in children and adolescents with Down's syndrome in a non–Caucasian population

Dear Participant:

We would like to invite you to voluntarily participate in this study: “Characteristics of type 1 diabetes mellitus in children and adolescents with Down's syndrome in a non-Caucasian population” which will evaluate clinical and epidemiological aspects of type 1 diabetes mellitus in children and adolescents with Down's syndrome in a non-Caucasian population and your medical information will be obtained from medical records.

Your name or other identifying information will not be used when discussing or reporting data, which guarantees your anonymity, and the results will be published in a way that does not identify the volunteers. You will not have to pay anything as a result of taking part in this study, and you will receive no payment of any kind, as the study involves only the collection of data from existing medical records

There is a minimal risk of loss of confidentiality or personal data as the researchers are committed to not revealing the identity of the patients, and using the information obtained for strictly scientific purposes.

We would like to explain that your participation is voluntary and you can refuse to participate or withdraw your consent and discontinue your participation, without affecting the care, services or benefits to which you are entitled.

If you accept to participate voluntarily in the study, you will be given a copy of this Informed Consent Form.

If you have any doubts or questions at any stage of the study, you can contact the researchers by phone – Débora Martins Ferreira Pessoa (11982051122), Nara Lívia Rezende Soares da Paz Oliveira (11984311038), or at the address: *Rua Cesário Mota Junior, 112, Vila Buarque, SP*. If you have any concerns or doubts about research ethics, please contact the Research Ethics Committee (CEP) – *Rua: Santa Isabel, 305, 4° andar – Fone: (11) 2176-7689 – E-mail: cepsc@santacasasp.org.br*.

I, ____________________________, have read this consent form (or it has been read to me), and I understand the information it provides about the study “Characteristics of type 1 diabetes mellitus in children and adolescents with Down's syndrome in a non–Caucasian population”.

I discussed the study with the researchers Débora Martins Ferreira Pessoa or Nara Lívia Rezende Soares and agreed to participate. I understand the purpose of the study, the risks and the guarantees of confidentiality. I also understand that I will not have to pay to participate in the study, and that I will receive no payment, that I have guaranteed access to hospital treatment when needed.

I voluntarily agree to participate in the study and I know I can withdraw my consent at any time, before or during it, without penalties or loss to my treatment at this hospital.

I authorize the use of my records, and any observations or findings made during the course of this study for education, publication and/or presentation.

Date:

São Paulo,

_____________________________________________

Signature of the participant, parent or legal guardian

_____________________________________________

Signature of the Researcher

## APPENDIX 3 ASSENT FORM

### Characteristics of type 1 diabetes mellitus in children and adolescents with Down's syndrome in a non–Caucasian population

I, ___________________________________________ received information and an explanation about the study: Characteristics of type 1 diabetes mellitus in children and adolescents with Down's syndrome in a non–Caucasian population. I am aware that my medical information will be obtained from my medical records.

I was also informed that participation in this study is voluntary and I do not have to take part. No one will be mad at me if I decide not to do this study and even if I start, I can stop at any time I want without making any difference to my treatment at this hospital.

I know I may ask questions about the study at any time, I can contact the researchers Débora Martins Ferreira Pessoa, (11) 982051122 or Nara Lívia Rezende Soares, (11) 984311038 or go to this address: Rua Cesário Mota Junior, 112, Vila Buarque – SP and ask my questions; and that if I have any concerns about research ethics I just go to or call the Research Ethics Committee (CEP) – *Rua: Santa Isabel, 305, 4° andar – Fone: (11) 2176-7689 – E-mail: cepsc@santacasasp.org.br.*

I declare that, after the researchers explained to the study to me, I agree to allow my participation in the study.

I understand that signing here means that I have read this form (or have had it read to me) and am willing to be in this study.

Date:

São Paulo,

_____________________________________________

Signature of the Participant

_____________________________________________

Signature of the Researcher
